# Prediction of hub genes and key pathways associated with the radiation response of human hematopoietic stem/progenitor cells using integrated bioinformatics methods

**DOI:** 10.1038/s41598-023-37981-6

**Published:** 2023-07-04

**Authors:** Yoshiaki Sato, Hironori Yoshino, Junya Ishikawa, Satoru Monzen, Masaru Yamaguchi, Ikuo Kashiwakura

**Affiliations:** 1 Department of Radiation Science, Hirosaki University Graduate School of Health Sciences, Hirosaki, Aomori 036-8564 Japan; 2 Department of Medical Radiologic Technology, Faculty of Health Sciences, Kyorin University, Mitaka, Tokyo 181-8612 Japan

**Keywords:** Computational biology and bioinformatics, Stem cells, Biomarkers

## Abstract

Hematopoietic stem cells (HSCs) are indispensable for the maintenance of the entire blood program through cytokine response. However, HSCs have high radiosensitivity, which is often a problem during radiation therapy and nuclear accidents. Although our previous study has reported that the combination cytokine treatment (interleukin-3, stem cell factor, and thrombopoietin) improves the survival of human hematopoietic stem/progenitor cells (HSPCs) after radiation, the mechanism by which cytokines contribute to the survival of HSPCs is largely unclear. To address this issue, the present study characterized the effect of cytokines on the radiation-induced gene expression profile of human CD34^+^ HSPCs and explored the hub genes that play key pathways associated with the radiation response using a cDNA microarray, a protein–protein interaction-MCODE module analysis and Cytohubba plugin tool in Cytoscape. This study identified 2,733 differentially expressed genes (DEGs) and five hub genes (TOP2A, EZH2, HSPA8, GART, HDAC1) in response to radiation in only the presence of cytokines. Furthermore, functional enrichment analysis found that hub genes and top DEGs based on fold change were enriched in the chromosome organization and organelle organization. The present findings may help predict the radiation response and improve our understanding of this response of human HSPCs.

## Introduction

Hematopoietic stem cells (HSCs) are the only cells that can self-renew and differentiate into the blood and immune cells throughout the lifetime of an organism. The homeostasis of HSCs, such as quiescence, proliferation, and differentiation is tightly regulated by their descendants, which mediate the secretion of cytokines in the bone marrow niche^1^, and cytokine stimulation is essential for maintaining hematopoietic homeostasis and survival after stress stimulation^2^. It is also known that the hematopoietic system is sensitive to oxidative stresses, such as radiation and chemotherapeutic agents^3^. For example, radiation exposure causes myelosuppression due to the lack of function of hematopoietic stem/progenitor cells (HSPCs) during radiotherapy and a nuclear disaster^4^. Moreover, recent reports have shown that HSPCs localize within the tumor microenvironment and facilitate tumor growth^5^. These reports suggest that it is important to understand the functional mechanism and reaction of HSPCs to stress stimuli. However, the effect of cytokines which is essential to regulate the function of HSPCs is not wholly understood.

Previous studies have shown that cytokine stimulation modulates cell fate^6–9^. For example. Zhou et al. showed that bone marrow adipocyte-mediated secretion of stem cell factor is important for promoting the regeneration of HSCs and hematopoiesis after irradiation^7^. Previously, we demonstrated that a combination of interleukin-3 (IL-3), stem cell factor (SCF), and thrombopoietin (TPO) significantly increased the number of viable irradiated cord blood CD34^+^ cells in comparison to other combinations^8^. Although an Ingenuity Pathway Analysis in our previous study predicted that Myc is an upstream regulator of the radiation-induced gene expression of human HSPCs in the presence or absence of the above combination of cytokines^9^, this is not considered to be the effect of the above combination of cytokines. Therefore, the mechanism by which cytokine stimulation modulates radiation responses in human HSPCs is still unclear.

Growing evidence has shown that various factors, including transcription factors, epigenetic regulatory factors, and metabolic regulators, are involved in the regulation of the stress response of HSPCs to radiation and infections^10–12^. However, since most studies related to HSPCs used mouse models, it is not unclear whether their findings can be extended to human HSPCs. Thus far, there is a lack of knowledge surrounding the response of human HSPCs to radiation.

Bioinformatics analyses and computational frameworks based on gene expression data are now widely used to identify hub genes that play an important role in a biological system^13–15^. This approach plays a major role in understanding the molecular function and protein–protein interactions (PPIs) in response to stimulation^16, 17^. For example, Su et al. revealed that psoriasis and atherosclerosis had many common pathogenic mechanisms that might be mediated by specific hub genes, through their microarray analysis of gene expression profiles^16^. In the present study, we conducted integrated bioinformatics analyses of PPIs with the data set of microarray analysis to predict hub genes that are associated with the radiation response in human HSPCs.

## Material and methods

### Collection and purification of placental/umbilical cord blood CD34^+^ cells

The study was approved by the Committee of Medical Ethics of Hirosaki University Graduate School of Medicine (2017-135, Hirosaki, Japan), and all experiments were performed by relevant guidelines and regulations. After informed consent was obtained from mothers, the placental/umbilical cord blood was collected at the end of full-term deliveries using a sterile collection bag containing the anticoagulant citrate–phosphate–dextrose, according to the guidelines of the Tokyo Cord Blood Bank (Tokyo, Japan). These samples were separately isolated and used for each experiment. Within 24 h after the collection of cord blood, the light-density mononuclear cord blood cells were separated by centrifugation on Limphosepar I (1.077 g/ml; Immuno-Biological Laboratories, Takasaki, Japan) for 30 min at 300*g* and washed three times with phosphate-buffered saline containing 5 mM ethylenediaminetetraacetic acid. The cells were then processed for CD34^+^ cell enrichment according to the manufacturer's instructions. The Indirect CD34 Micro Beads Kit and an autoMACS Pro Separator (Miltenyi Biotec, Tokyo, Japan) were used for the positive selection of the CD34^+^ cells.

### In vitro irradiation and liquid culture

CD34^+^ cells were cultured in vitro after being exposed to X-rays. Thereafter, 5–7.5 × 10^5^ CD34^+^ cells were suspended in serum-free Iscove's modified Dulbecco's medium (Gibco^®^, Invitrogen, California, USA) supplemented with BIT9500 (StemCell Technologies Inc, Vancouver, Canada), a serum substitute for serum-free culture. Cell suspensions with cell densities of 2.0–3.0 × 10^5^ cells/ml were equally divided into five wells of a 24-well plate, and these cells were subjected to different conditions as follows: two of the cell fractions were irradiated with 2 Gy X-rays (150 kVp, 20 mA) using a MBR-1520R (Hitachi Medical Co., Tokyo, Japan) with a dose rate of approximately 1 Gy/min. Then, one of these cell fractions was cultured for 6 h with recombinant human IL-3 (Biosource, Tokyo, Japan), recombinant human SCF (PeproTech, Rocky Hill, New Jersey, USA), and recombinant human TPO (PeproTech, Rocky Hill, New Jersey, USA), and the other cell fraction was cultured without cytokines. If there is no cytokine stimulation, the proliferative capacity of hematopoietic stem cells will rapidly disappear^2^. This combination of cytokines applied in this study has been reported to be optimal for HSPCs survival and proliferation^8, 18^. The next two cell groups were not irradiated and were cultured with or without a combination of cytokines. The remaining cell group was directly used for the next stage of analysis.

### Microarray procedure

The total RNA was extracted from each of the five above-mentioned cell groups as described in a previous report^19^. The total RNAs were extracted using the RNeasy^®^ Micro Kit (Qiagen, Bothell, Washington, USA); their concentration and purity were determined using a bioanalyzer (Agilent Technologies, Santa Clara, California, USA) according to the manufacturer's instructions. The gene expression was analyzed using a GeneChip^®^ system with a Human Genome U133-plus 2.0 array (Affymetrix, Santa Clara, California, USA) according to the manufacturer's instructions. The scanned chip was analyzed using the GeneChip^®^ Analysis Suite software program Affymetrix) and obtained hybridization intensity data.

### Identification of differentially expressed genes

We applied the Limma R package to identify differentially expressed genes (DEGs) between the non-irradiated and irradiated samples using the “model.matrix”, “lmFit”, “eBays” and “topTable” functions^20^. The statistical significance threshold was set at log2(fold change, FC) > 1 or log2(FC) <  − 1 with Benjamini-Hochberg's adjusted p-value (adj.P.Val) < 0.05. Fisher's exact test was applied for the analysis of the effect of cytokine on the number of radiation-induced DEGs by the “fisher.test” function in R (v2.6.1). The DEGs were visualized in a volcano plot and heat map using the R package “ggplot2”. We performed agglomerative hierarchical clustering using Euclidean distance as the distance metric and complete linkage. Clustering was implemented in the R statistical environment.

### Gene ontology analysis, pathway enrichment Analysis, gene set enrichment analysis

The gene ontology (GO) and Kyoto Encyclopedia of Genes and Genomes (KEGG) pathway enrichment analysis for up- and down-regulated DEGs were performed using g:GOSt in g:Profiler^21^. The GO considers three aspects of gene function including molecular function (MF), cellular component (CC), and biological process (BP). The bubble diagrams were then created by the R package “ggplot2” to exhibit the top 5 of the GO terms and KEGG pathways based on the adjusted p-value. We used the tool Cytoscape version 3.9.1 (accessed on 17 July 2022) for the formation of an integrative network of key genes ^22^. For gene set enrichment analysis (GSEA), The Broad Institute provided the publicly available software^23, 24^. GSEA was performed with gene sets of chromosome organization and organelle organization from g:Profiler. The statistical significance of the false discovery rate (FDR) was set to 0.25, as recommended by the GSEA developer Broad Institute.

### Protein–protein network screening, hub genes identification, and module network construction

The online program STRING version 1.7.0 (accessed on 17 July 2022) was used to extract interconnected genes to create a network of PPI^25^. To visualize PPI, we applied the tool Cytoscape^22^. To identify significant genes in the subnetwork, a Cytoscape plugin Molecular Complex Detection (MCODE) version 2.0.2 was implemented with the parameters K-score (2), degree cutoff score (2), node cutoff score (0.2), and 100 maximum depths. To find the most intersected hub genes and modules, the Cytohubba plugin tool was used in Cytoscape, and the PPI-MCODE modules were also merged^26^. We used Cytohubba by five topological analysis methods such as Betweenness Centrality, Closeness Centrality, Degree, Stress, and Bottleneck were also added to the network for evaluating and selecting hub genes and modules. Information on the involvement of genes in clusters and top-score genes in the topological analysis is shown as a heat map using the R package “ComplexHeatmap”^27^. The intersection size between each gene set scored by each algorithm was shown as an upset plot using the R package “complex UpsetR”^28, 29^.

### Ethics statement

The study was approved by the Committee of Medical Ethics of Hirosaki University Graduate School of Medicine (2017-135, Hirosaki, Japan).

## Results

### Effect of radiation on the gene expression profiles in human HSPCs in the presence or absence of cytokines and the characterization of gene expression profiles

**Figure 1:**
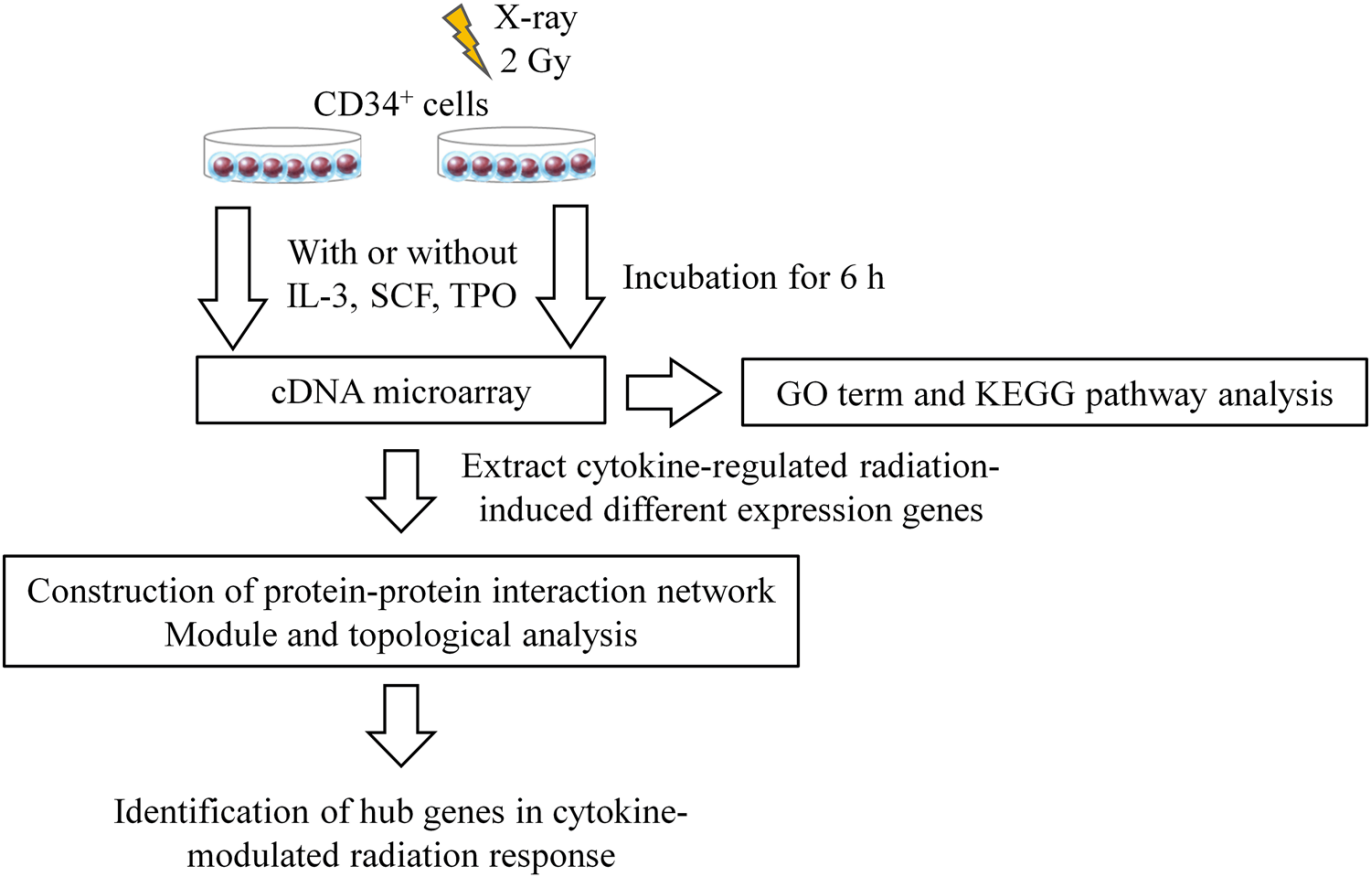
The flowchart of the methodology of identification of hub genes that modulate radiation response.

To predict the hub gene which plays a key role in radiation response in the presence of cytokine, this study re-analyzed the previous our data set of a cDNA microarray for human CD34^+^ HSPCs in the presence or absence of cytokines (IL-3, SCF, TPO)^9^, and integrated with module analysis and topological analysis. The overall workflow of this study is shown in Fig. 1.

**Figure 2:**
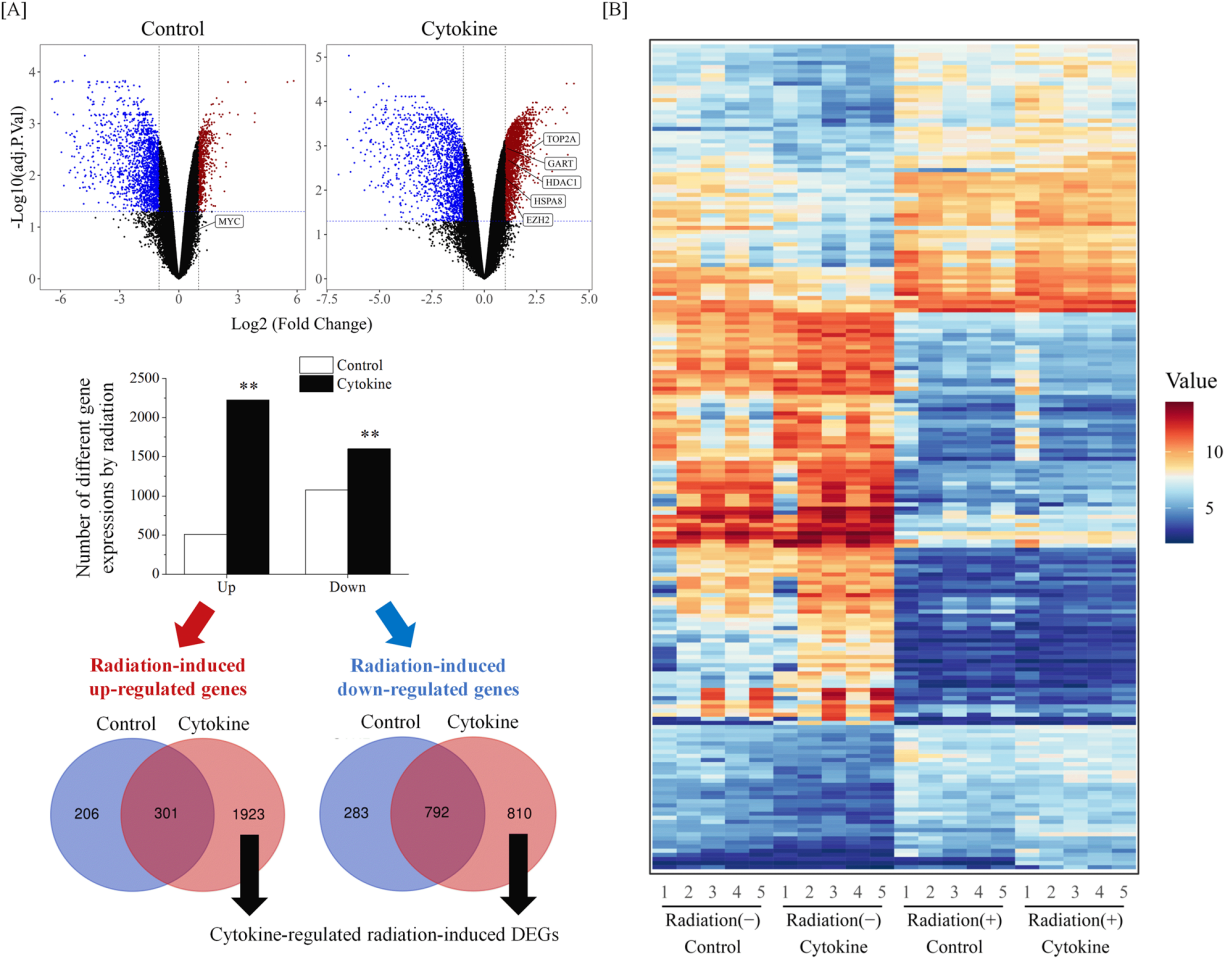
Effects of cytokine on radiation-induced gene expression profile. (**A**) (Top) Volcano plot showing DEGs by radiation. The log2FC value > 1 or log2FC value <  − 1 with adj.P.Val < 0.05 are the cutoff value for significant up-regulated (red color), non-significant (black color), and down-regulated (blue color) DEGs. (Middle) The number of DEGs by radiation in control and cytokine-treated cells is shown. **p < 0.01 (Fisher's exact test). (Bottom) Venn diagrams of genes exhibiting either up- or down-regulated by radiation in control and cytokine-treated cells. Labeled genes were identified as important regulators of radiation response in a previous our study or following analysis in this study. (**B**) Microarray heat map for normalized intensities of the top 100 up- and down-regulated genes.

The microarray analysis revealed a total of 1582 differentially expressed genes (DEGs) upon radiation exposure in the absence of cytokines, including 507 up-regulated and 1075 down-regulated DEGs (Fig. 2A, top and middle). We also observed a total of 3826 DEGs upon radiation exposure in the presence of cytokines, including 2224 up-regulated and 1602 down-regulated DEGs, as represented in the volcano plot (Fig. 2A, top and middle). The top 10 up- and down-regulated DEGs based on the FC in the presence of cytokines are presented in Table 1. The normalized intensities of the top 100 up- and down-regulated DEGs based on the FC are presented as a heat map in Fig. 2B. We observed more DEGs upon radiation exposure in the presence of cytokines than in their absence (Fig. 2A, middle). As expected, a Venn diagram revealed that most of the DEGs belonged to cytokine-treated cells, as 86% (1923/2430) of up-regulated DEGs and 43% (810/1885) (the largest group) of down-regulated DEGs were detected in cytokine-treated cells (Fig. 2A, bottom and Supplementary Table 1).

**Table 1:** TOP10 up-regulated and down-regulated genes in cytokine-treated cells.

Up-regulated			Down-regulated		
Gene symbol	Log(FC)	adj.P.Val	Gene symbol	Log(FC)	adj.P.Val
PRG2	4.27549016	3.95E−05	OLR1	−6.952132483	0.004448952
CLC	3.983691528	0.001593824	SERPINA1	−6.534179759	0.000722542
NUMA1	3.944307097	0.000181969	IGSF6	−6.459056458	9.25E−06
MPO	3.926619853	3.95E−05	PID1	−6.388781353	0.000174328
ELANE	3.866657812	0.000135446	SASH1	−6.388582451	5.35E−05
SCN3A	3.76442298	0.000151688	CCL20	−6.380188729	0.003871562
IGLL1	3.402116117	0.00031661	IL1RN	−6.260062428	0.001108947
PRTN3	3.247495001	0.000151992	CXCL1	−6.195853713	0.002422895
TMEM156	3.23649267	0.00377058	MS4A7	−6.017928677	7.87E−05
MS4A3	3.13746366	0.000135446	SMPDL3A	−5.96387065	4.69E−04

**Figure 3:**
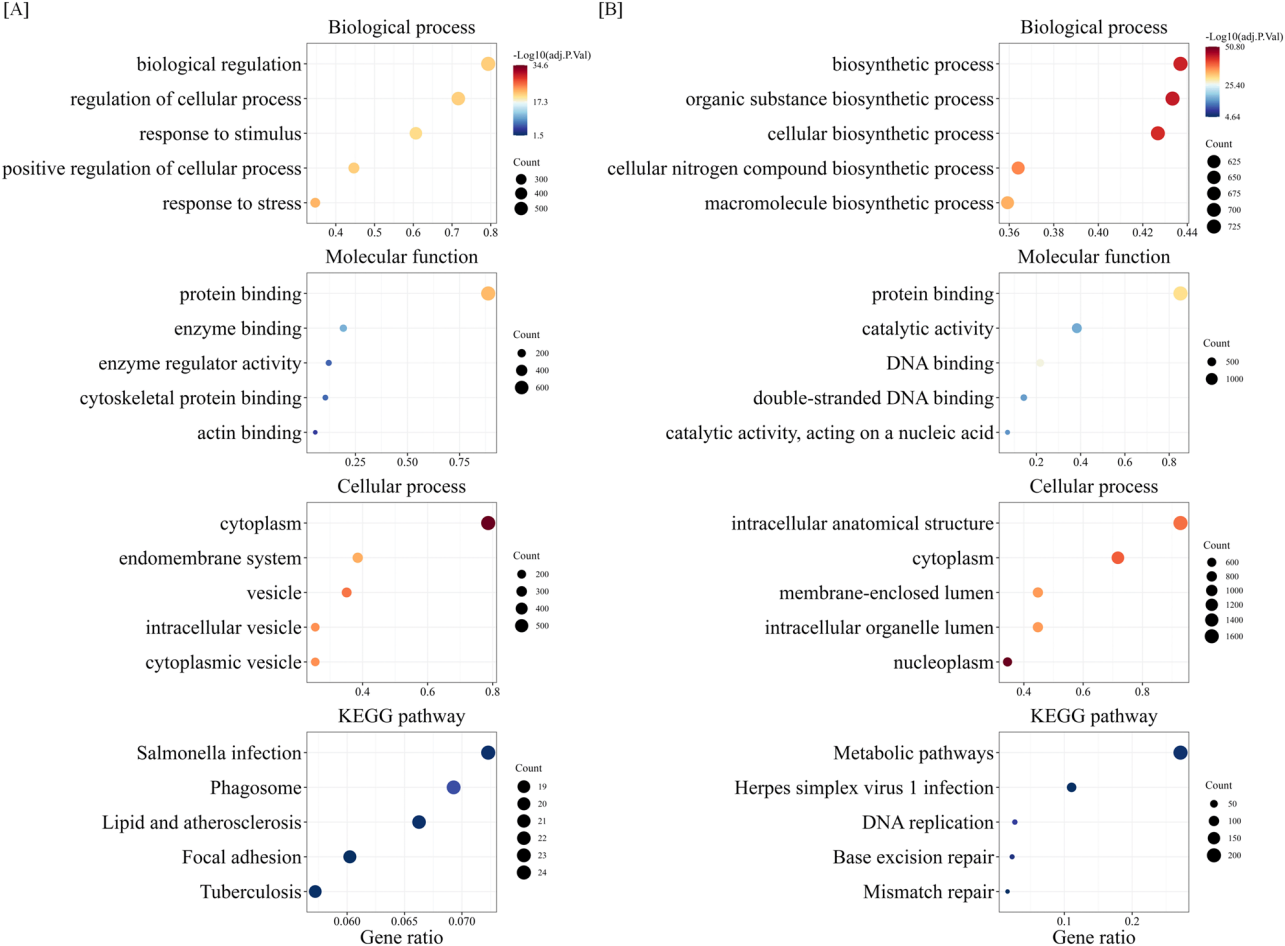
GO and KEGG functional enrichment analysis with cytokine-regulated (**A**) down- or (**B**) up-regulated DEGs. GO functional annotation and KEGG pathway analysis for DEGs. Bubble plots of the top 5 terms and pathways in biological process, cellular component, molecular function, and KEGG pathway analysis based on adjusted p-value are shown. Gene ratio is defined as the ratio between intersection size and query size. The number refers to interaction size, i.e., the number of genes corresponding to an ontology term.

Since cytokines modulate the gene expression profile in irradiated cells (Fig. 2), we hypothesized that examining the cytokine-regulated radiation-induced DEGs would improve our understanding of the role and function of HSPCs in response to radiation. To gain insight into the functional significance of cytokine-regulated radiation-induced DEGs, we conducted GO and KEGG pathway analyses (Fig. 3).

As shown in Fig. 3A and Supplementary Table 2, cytokine-regulated down-regulated DEGs were enriched GO BP terms related to response to stress; response to stimulus; regulation of cellular process; positive regulation of cellular process; and biological regulation. In the CC group, cytokine-regulated down-regulated DEGs were enriched in vesicle; intracellular vesicle; endomembrane system; cytoplasmic vesicle; and cytoplasm. The most significant MF for down-regulated DEGs involved protein binding, enzyme regulator activity, enzyme binding, cytoskeletal protein binding and actin binding. The most significant cytokine-regulated down-regulated DEGs in the KEGG pathway analysis were involved in tuberculosis including BID and MALT1; salmonella infection including ACTR3 and CYTH1; phagosomes including CYBA and ATP6V0B; lipids and atherosclerosis including BID and CYBA; and focal adhesion including MYL12A and FN1.

On the other hand, cytokine-regulated up-regulated DEGs were enriched GO BP terms related to organic substance biosynthetic process; biosynthetic process; cellular biosynthetic process; macromolecule biosynthetic process; and cellular nitrogen compound biosynthetic process. In the CC group, cytokine-regulated up-regulated DEGs were enriched nucleoplasm; membrane-enclosed lumen; intracellular organelle lumen; intracellular anatomical structure; and cytoplasm. The most significant MF for cytokine-regulated up-regulated DEGs were involved in ion binding; double-stranded DNA binding; DNA binding; catalytic activity; acting on a nucleic acid; and catalytic activity. The most significant cytokine-regulated up-regulated DEGs under the KEGG pathway analysis were involved in mismatch repair including MSH6 and POLD2, metabolic pathways including GCSH and PFKP, herpes simplex virus 1 infection including ZNF510and ZNF83, DNA replication and base excision repair including POLE3 and POLD2 (Fig. 3B and Supplementary Table 2). The above results indicated that the DEGs were mostly associated with cell survival such as biosynthetic processes and the damage repair pathway.

### Identification of hub gene by PPI network and modular analyses

**Figure 4:**
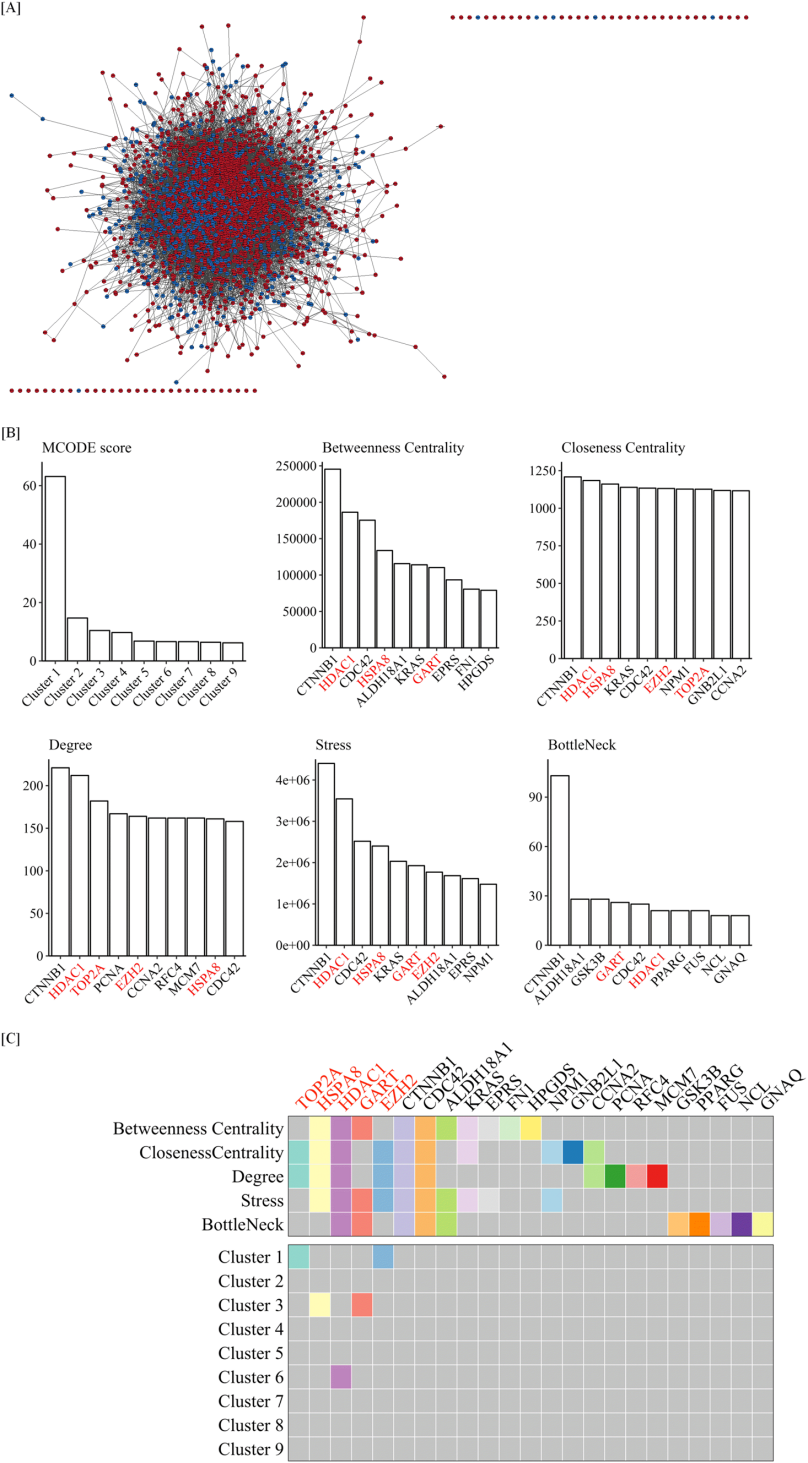
Module and topological analysis. (**A**) PPIs with cytokine-regulated radiation-induced DEGs were analyzed using the STRING database. Clusters including hub genes are shown. Up-regulated DEGs are represented by red color nodes and down-regulated DEGs are represented by blue color nodes. Nodes, shown as the circle in the figure, represent DEGs. Edges, which connect to signature nodes in the figure, represent interactions between nodes. (**B**) Scores of analyses by MCODE, Betweenness Centrality, Closeness Centrality, Degree, Stress, and Bottleneck are presented. The red font color indicates hub genes. (**C**) The genes identified in analyses by MCODE, Betweenness Centrality, Closeness Centrality, Degree, Stress, and Bottleneck are shown as a heat map. The red font color indicates hub genes. A filled color block indicates the genes are involved in the cluster or scored highly in the topological analysis.

Since GO analysis showed that cytokine-regulated radiation-induced DEGs were mostly associated with cell survival, all cytokine-regulated radiation-induced DEGs were analyzed using the STRING database to identify the important PPIs for cell survival after radiation. We constructed a PPI network with these data using the Cytoscape software program and the result showed that the network has 2550 nodes and 28,388 edges (Fig. 4A). Furthermore, we applied the MCODE algorithm to the constructed network. This method found nine clustering modules with high MCODE scores (> 6; Fig. 4B and Supplementary Fig. 1A–I). The top-scoring module was Cluster 1, which consisted of 73 nodes and 2273 edges with a score of 63.139. Cluster 2 included 85 nodes and 617 edges with a score of 14.69, and Cluster 3 consisted of 67 nodes and 344 edges with a score of 10.424. Cluster 4 had 16 nodes and 73 edges with a score of 9.733. Cluster 5 had 95 nodes and 319 edges with a score of 6.787. Cluster 6 had 56 nodes and 182 edges with a score of 6.618. Cluster 7 had 11 nodes and 33 edges with a score of 6.6. Cluster 8 also had 11 nodes and 32 edges with a score of 6.4. Cluster 9 also had 11 nodes and 31 edges with a score of 6.2. All clusters are shown in Supplementary Fig. 1 and Supplementary Tables 3.

Microarray analyses of gene expression profiles have been used to identify potential hub genes in many diseases, and this method is useful for identifying biomarker and therapeutic targets^30, 31^. Therefore, to identify hub genes that might play key roles in the cytokine-mediated modulation of the radiation response, we further analyzed all cytokine-regulated DEGs with five topological analysis methods. The present study applied four Global-based methods which examine the relationship between the node and the entire network, and one considers the relationship between the node and its direct neighbors^29^. These algorisms showed that the top 10 genes scored highly according to each topological analysis method (Fig. 4B and Supplementary Table 3). Among the Global-based methods including Betweenness Centrality, Closeness Centrality, Stress, and Bottleneck, Closeness Centrality shared five of ten top genes with the Local-based method Degree, and Bottleneck had five unique genes in the top 10 genes (Supplementary Fig. S1J). To predict the hub genes, we defined the hub genes as a gene located in the clustering module with a high MCODE score and ranked in the top 10 in any topological analysis. This method identified five unique hub genes, including Transcription-associated topoisomerase 2α (TOP2A), Enhancer of zeste homolog 2 (EZH2), Heat shock 70 kDa protein 8 (HSPA8), phosphoribosylglycinamide formyltransferase (GART) and Histone deacetylase 1 (HDAC1) (Table 2). 3 clusters included hub genes (Fig. 4C, top): Cluster 1 includes TOP2A and EZH2; Cluster 3 includes HSPA8 and GART; and Cluster 6 includes HDAC1. Therefore, these results suggest that the gene network regulated by these hub genes may be considered a major gene driver and likely to play a significant role in the radiation response by cytokine treatment.

**Table 2:** List of hub genes.

Gene symbol	HGNC	Log(FC)	adj.P.Val
TOP2A	HGNC:11989	2.317158787	0.001118789
HSPA8	HGNC:5241	1.010998768	0.005391001
HDAC1	HGNC:4852	1.116904428	0.00204618
GART	HGNC:4163	1.041735941	0.00112017
EZH2	HGNC:3527	1.247718132	0.013127787

### The association of hub genes and cytokine-regulated radiation-induced DEGs network with the biological process of human HSPCs in response to radiation

**Figure 5:**
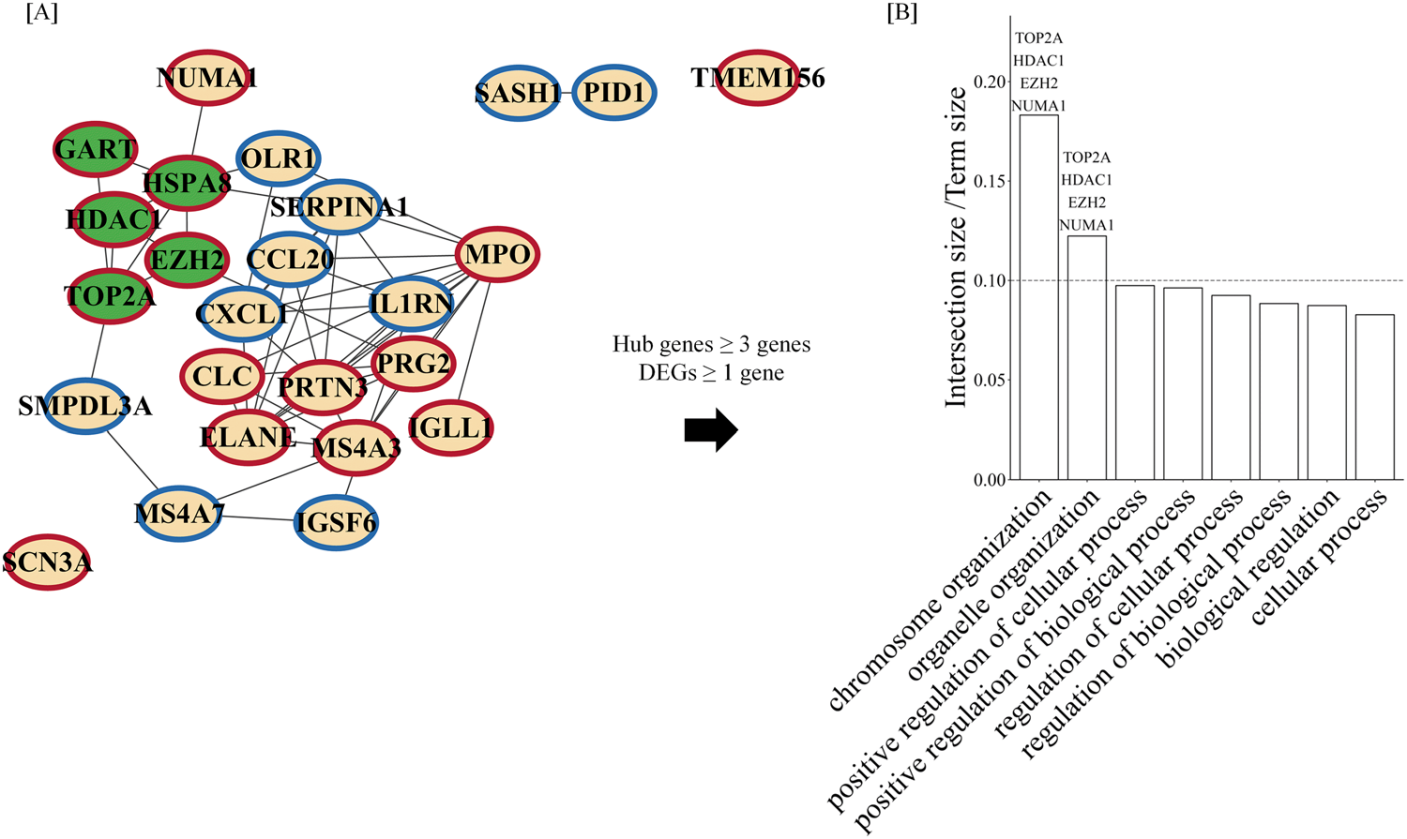
Biological process contributed by hub genes and top cytokine-regulated radiation-induced DEGs. (**A**) PPIs with hub genes and the top 10 cytokine-regulated radiation-induced DEGs were analyzed using the STRING database. Nodes of hub genes and DEGs were filled in green and pale orange, respectively. (**B**) The ratios between intersection size and term size are present. The number refers to interaction size, i.e., the number of genes corresponding to an ontology term. The gene symbols on the bar plot indicate the gene is included in the term.

To investigate the association of hub genes with cytokine-regulated radiation-induced DEGs, we constructed a PPI network with hub genes and the top 10 cytokine-regulated radiation-induced DEGs based on FC (Fig. 5A). The results showed that some hub genes interacted with DEGs. Finally, we investigated the contribution of hub genes and cytokine-regulated radiation-induced DEGs to the radiation response in human HSPCs and found that among BP enriched in up-regulated cytokine-regulated DEGs, eight BP term names including “chromosome organization” and “organelle organization” include at least three hub genes and one up-regulated DEG ranked in top 10 (Fig. 5B). The hub genes are TOP2A, HDAC1, and EZH2 and DEG is Nuclear mitotic apparatus protein 1 (NUMA1) was included in these terms. The intersection size of cytokine-regulated radiation-induced DEGs is higher than 10% in these BP term sizes. In addition, GSEA showed that the genes related to these terms were more enriched in the group treated by radiation with cytokine than that without cytokine (Supplementary Fig. 3). Therefore, this result may suggest that cytokine treatment modulates the “chromosome organization” and “organelle organization” of human HSPCs in response to radiation.

## Discussion

Radiation induces genotoxic stress, and irradiated cells engage in diverse biological responses, including DNA repair, cell-cycle arrest, and cell death, to maintain tissue/cellular homeostasis^32^. As expected, genes commonly up-regulated by radiation in both control and cytokine-treated cells were enriched in the cell cycle and DNA replication in this study (Supplementary Fig. 2B). Results showed that 206 up-regulated DEGs that were only detected in control cells were concentrated in a few GO terms and pathways; this suggests that cytokine-nonresponsive radiation-responsive genes may be uncoordinated (Fig. 2A and Supplementary Fig. 2). Interestingly, these genes were enriched BP terms non-coding RNA (ncRNA) processing and ncRNA metabolic process (Supplementary Fig. 2D). Once thought of as arising from “junk DNA”, growing evidence supports that ncRNAs are key molecules in cellular processes and responses to stress, such as DNA damage^33^. Several studies have reported that ncRNA is involved in the regulation of radiosensitivity, but the roles of ncRNA in the radiation response remain to be elucidated. The present results suggest that ncRNAs may be involved in the radiation response of HSPCs.

It is known that radiation causes immunosuppressive responses^34^. In this study, commonly down-regulated DEGs were concentrated in the BP terms such as immune response, immune system process, and response to stimuli (Supplementary Fig. 2A, C), and cytokine-regulated down-regulated DEGs were enriched in tuberculosis and salmonella infection (Fig. 3A). Several studies have shown that the activation of the immune response protects several types of cells from radiation^35, 36^. For example, Leibowitz et al., have reported that radiation-inducible interferon-β production in the niche couples intestinal stem cells injury and regeneration^35^. Therefore, these results may reflect that radiation causes dysfunction of the immune response, and improvement from the dysfunction of the immune response could be an effective target to protect HSPCs from radiation. On the other hand, cytokine-regulated up-regulated DEGs were enriched in “Herpes simplex virus 1 infection” in Fig. 3. In this term, 73 of 82 genes are the zinc finger (ZNF) gene family. ZNF is well-known as a transcriptional factor that recognizes and binds sequence-specific DNA^37^. Previously, one of the ZNF proteins, ZFP521 has been involved in hematopoietic stem/progenitor cell differentiation^38^. Moreover, some of the ZNF genes have been reported as the possible radiosensitivity biomarker of breast cancer^39^. However, little study shows the role of the ZNF family on radiation response and/or human HSPCs. Therefore, these results, with very little information on the radiation response of human HSPCs, may provide a stepping stone to studying the novel role of the ZNF family.

The present study found 5 hub genes (TOP2A, EZH2, HSPA8, GART, HDAC1) that play key roles in the cytokine-mediated modulation of the radiation response (Fig. 4). These genes are involved in the stress response, such as DNA repair and metabolic pathways. TOP2A is a type II topoisomerase and a nuclear enzyme involved in chromosome condensation as well as relief of torsional stress during DNA transcription, replication, and DNA repair^40–42^. Recently, TOP2A was detected as a component of the DNA double-strand break (DSB) repair complex^43^. Lei et al. reported the interaction of TOP2A and transglutaminase 2 to promote DNA damage repair in lung cancer cells after exposure to ionizing radiation^43^. It has been also reported that TOP2A is involved in radioresistance^44, 45^. These previous studies suggest that cytokine treatment after radiation exposure may lead to TOP2A-mediated DNA repair in HSPCs.

EZH2 encodes a histone methyltransferase, which is the catalytic core protein of the polycomb repressor complex 2, which silences target genes^46, 47^. EZH2 provides proliferative advantages to eukaryotic cells through interaction with pathways of key elements that control cell growth arrest, differentiation, and DNA repair^48, 49^. Zhang et al. reported that EZH2 promotes the process of radiation-induced stem cell-like transformation and radio-resistance through DNA damage repair and autophagy^48^. Since EZH2 has been reported to directly induce a group of DNA repair genes^50^, and cytokine-regulated up-regulated DEGs were enriched in pathways related to DNA repair, cytokine treatment after radiation exposure may contribute to DNA repair through chromatin remodeling by EZH2.

HSPA8 is a member of the heat shock protein family, the main function of which is to act as chaperone proteins by repairing misfolded proteins under various stress conditions and degradation of proteins^51, 52^. It is reported that misfolded proteins impair stem cell quiescence and self-renewal by overwhelming the proteasome and disrupting protein homeostasis^53^. Radiation has been reported to increase protein misfolding through the generation of reactive oxygen species^54^, and our results suggest that cytokine treatment after radiation exposure may maintain protein homeostasis of HSPCs against induction of misfolded protein by radiation.

GART is a critical enzyme of purine synthesis that is involved in nucleotide synthesis pathways^55^. Purine synthesis reportedly plays a critical role in DNA repair and radioresistance^56–58^. Karigane et al. also reported that purine metabolism is crucial for initiating HSPCs proliferation during stress hematopoiesis^58^. In support of this, KEGG analysis showed that the metabolic pathway which includes Purine metabolism and Nucleotide metabolism, and this term was observed in only the presence of cytokines., Pyrimidine metabolism (Supplementary Table 4). Therefore, our results may indicate that cytokine treatment after radiation exposure leads to appropriate stress response to radiation in HSPCs.

HDAC1 is a histone deacetylase that removes acetyl groups from lysine residues. HDAC1 has been reported to participate in the DNA damage response^59^. Miller et al. showed that HDAC1/2 was rapidly recruited to DNA damage sites to promote histone H3 lysine 56 hypo-acetylation, and HDAC1/2-depletion confers sensitivity to DNA-damaging agents due to an error in the DSB repair, particularly in the non-homologous end-joining pathway^60^. Many studies have demonstrated that HDAC1 plays key roles in DNA repair in irradiated cells and may be an effective target for promoting the effect of radiotherapy^60^. Thus, in addition to EZH2, HDAC1 may also play a key role in the DNA damage response in irradiated cells, and cytokine treatment after radiation exposure may support the survival of irradiated HSPCs through chromatin remodeling in HSPCs.

The biological target of ionizing radiation is DNA. Interestingly, cytokine treatment upregulated genes involved in DNA-related pathways, such as the GO terms of double-stranded DNA binding and DNA binding, and has been reported to be involved in chromatin modification and DNA repair (Fig. 3B). In addition, some of the hub genes (TOP2A, HDAC1, and EZH2) and the top 10 cytokine-regulated DEGs (NUMA1) enriched chromosome organization (Fig. 5B), and related genes were more enriched in the group treated by radiation with cytokine than that without cytokine (Supplementary Fig. 3), suggesting that cytokine treatment modulates the gene expression pattern by inducing the hub genes and leading to the differential pathways (chromosome and organelle organization) rather than simply enhancement or weakening in the gene expression level response to radiation. DNA binding and chromatin modification and organization are closely related and significantly affect gene expression through the accessibility of DNA-binding proteins, leading to different responses to stimuli^61^. However, while some studies have reported on the importance of other areas in stress responses, such as the mitochondria and endoplasmic reticulum^62, 63^, the findings of the present study suggest the importance of investigating events around DNA to reveal the key factors involved in modulating the radiation response.

The present study was associated with some limitations. For example, we collected samples only 6 h after radiation exposure, as the number of human CD34^+^ HSPCs obtained from each cord blood sample was small. In addition, since our previous reports showed that the survival fraction and clonogenic potential of HSPCs rapidly decreased by 12 h after irradiation^2, 64^, we assumed a dramatic alteration in the gene expression occurred within 6 h after radiation exposure. Furthermore, the study of radiation effects on HSPCs, which are capable of self-renewal and rapidly lose their properties without the necessary stimuli, has many limitations; most of the findings on DNA repair pathways have been studied in cultured cells, etc. Detailed evaluation in more appropriate experimental systems is required in the future. Although HSPCs have been shown to have high radiosensitivity and be indispensable for hematopoietic homeostasis, the types of gene expression and pathway alternations that determine the radiation response of human HSPCs have been unclear. Therefore, the present study may improve our understanding of the radiation response of HSPCs and contribute to the development of biological markers for hematopoietic disorders after irradiation.

## Conclusion

This study predicted TOP2A, EZH2, HSPA8, GART, and HDAC1 as hub genes associated with the cytokine-mediated radiation response. Furthermore, “chromosome organization” and “organelle organization” were key pathways in characterizing the irradiated human HSPCs in the presence of cytokine. Although further studies are needed to address the role of these hub genes in the radiation response of human CD34^+^ HSPCs, the present findings may help predict the radiation response and improve our understanding of this response regarding human HSPCs.

## Supplementary Information

Supplementary Figures.

Supplementary Table 1.

Supplementary Table 2.

Supplementary Table 3.

Supplementary Table 4.

## Data Availability

The datasets generated and analyzed during the current study are available from the corresponding author upon reasonable request.

## References

[1] Lu Y, Hu M, Zhang Z, Qi Y, Wang J (2020). The regulation of hematopoietic stem cell fate in the context of radiation. Radiat. Med. Protect..

[2] Ishikawa J, Hayashi N, Yamaguchi M, Monzen S, Kashiwakura I (2015). Characteristics of human CD34+ cells exposed to ionizing radiation under cytokine-free conditions. J. Radiat. Res..

[3] Kato K, Omori A, Kashiwakura I (2013). Radiosensitivity of human haematopoietic stem/progenitor cells. J. Radiol. Prot..

[4] Kim J-H (2014). NRF2-mediated Notch pathway activation enhances hematopoietic reconstitution following myelosuppressive radiation. J. Clin. Invest..

[5] Parsons TM (2022). Intratumoural haematopoietic stem and progenitor cell differentiation into M2 macrophages facilitates the regrowth of solid tumours after radiation therapy. Br. J. Cancer.

[6] Mendelson A, Frenette PS (2014). Hematopoietic stem cell niche maintenance during homeostasis and regeneration. Nat. Med..

[7] Zhou BO (2017). Bone marrow adipocytes promote the regeneration of stem cells and haematopoiesis by secreting SCF. Nat. Cell Biol..

[8] Kashiwakura I (2006). Regeneration of megakaryocytopoiesis and thrombopoiesis in vitro from X-irradiated human hematopoietic stem cells. Radiat. Res..

[9] Tsujiguchi T (2016). Expression analysis of radiation-responsive genes in human hematopoietic stem/progenitor cells. J. Radiat. Res..

[10] Tung LT (2021). p53-dependent induction of P2X7 on hematopoietic stem and progenitor cells regulates hematopoietic response to genotoxic stress. Cell Death Dis..

[11] Sun X (2021). Nicotinamide riboside attenuates age-associated metabolic and functional changes in hematopoietic stem cells. Nat. Commun..

[12] Demel UM (2022). A complex proinflammatory cascade mediates the activation of HSCs upon LPS exposure in vivo. Blood Adv..

[13] Fiscon G, Conte F, Licursi V, Nasi S, Paci P (2018). Computational identification of specific genes for glioblastoma stem-like cells identity. Sci. Rep..

[14] Li M (2021). Identification and clinical validation of key extracellular proteins as the potential biomarkers in relapsing–remitting multiple sclerosis. Front. Immunol..

[15] Nangraj AS (2020). Integrated PPI- and WGCNA-retrieval of hub gene signatures shared between Barrett’s esophagus and esophageal adenocarcinoma. Front. Pharmacol..

[16] Su W (2021). Exploring the pathogenesis of psoriasis complicated with atherosclerosis via microarray data analysis. Front. Immunol..

[17] Grimaldi AM (2020). The new paradigm of network medicine to analyze breast cancer phenotypes. Int. J. Mol. Sci..

[18] Kashiwakura I (2003). Protective effects of thrombopoietin and stem cell factor on X-irradiated CD34+ megakaryocytic progenitor cells from human placental and umbilical cord blood. Radiat. Res..

[19] Kato K (2010). Relationship between radiosensitivity and Nrf2 target gene expression in human hematopoietic stem cells. Radiat. Res..

[20] Ritchie ME (2015). limma powers differential expression analyses for RNA-sequencing and microarray studies. Nucleic Acids Res..

[21] Reimand J (2019). Pathway enrichment analysis and visualization of omics data using g:Profiler, GSEA, Cytoscape and EnrichmentMap. Nat. Protoc..

[22] Shannon P (2003). Cytoscape: A software environment for integrated models of biomolecular interaction networks. Genome Res..

[23] Subramanian A (2005). Gene set enrichment analysis: A knowledge-based approach for interpreting genome-wide expression profiles. Proc. Natl. Acad. Sci..

[24] Mootha VK (2003). PGC-1α-responsive genes involved in oxidative phosphorylation are coordinately downregulated in human diabetes. Nat. Genet..

[25] Saito R (2012). A travel guide to Cytoscape plugins. Nat. Methods.

[26] Chin C-H (2014). cytoHubba: Identifying hub objects and sub-networks from complex interactome. BMC Syst. Biol..

[27] Gu Z, Eils R, Schlesner M (2016). Complex heatmaps reveal patterns and correlations in multidimensional genomic data. Bioinformatics.

[28] Lex A (2014). UpSet: Visualization of intersecting sets. IEEE Trans. Vis. Comput. Graph..

[29] Krassowski, M. *R package Version 0.5*. Vol. 18 (2020).

[30] Al Mehedi Hasan M, Maniruzzaman M, Shin J (2022). Identification of key candidate genes for IgA nephropathy using machine learning and statistics based bioinformatics models. Sci. Rep..

[31] Wu B, Xi S (2021). Bioinformatics analysis of differentially expressed genes and pathways in the development of cervical cancer. BMC Cancer.

[32] Swift LH, Golsteyn RM (2014). Genotoxic anti-cancer agents and their relationship to DNA damage, mitosis, and checkpoint adaptation in proliferating cancer cells. Int. J. Mol. Sci..

[33] May JM, Bylicky M, Chopra S, Coleman CN, Aryankalayil MJ (2021). Long and short non-coding RNA and radiation response: A review. Transl. Res..

[34] Weichselbaum RR, Liang H, Deng L, Fu Y-X (2017). Radiotherapy and immunotherapy: A beneficial liaison?. Nat. Rev. Clin. Oncol..

[35] Leibowitz BJ (2021). Interferon b drives intestinal regeneration after radiation. Sci. Adv..

[36] Burdelya LG (2013). Central role of liver in anticancer and radioprotective activities of Toll-like receptor 5 agonist. Proc. Natl. Acad. Sci..

[37] Cassandri M (2017). Zinc-finger proteins in health and disease. Cell Death Discov..

[38] Liu C (1999). SZF1: A novel KRAB-zinc finger gene expressed in CD34+ stem/progenitor cells. Exp. Hematol..

[39] Yan D (2021). Developing ZNF gene signatures predicting radiosensitivity of patients with breast cancer. J. Oncol..

[40] Pommier Y, Sun Y, Huang SN, Nitiss JL (2016). Roles of eukaryotic topoisomerases in transcription, replication and genomic stability. Nat. Rev. Mol. Cell Biol..

[41] Nielsen CF, Zhang T, Barisic M, Kalitsis P, Hudson DF (2020). Topoisomerase IIα is essential for maintenance of mitotic chromosome structure. Proc. Natl. Acad. Sci..

[42] Reymer A, Zakrzewska K, Lavery R (2018). Sequence-dependent response of DNA to torsional stress: A potential biological regulation mechanism. Nucleic Acids Res..

[43] Lei X (2021). Nuclear transglutaminase 2 interacts with topoisomerase II⍺ to promote DNA damage repair in lung cancer cells. J. Exp. Clin. Cancer Res..

[44] Zhang Y (2022). TOP2A correlates with poor prognosis and affects radioresistance of medulloblastoma. Front. Oncol..

[45] Simon JA, Lange CA (2008). Roles of the EZH2 histone methyltransferase in cancer epigenetics. Mutat. Res./Fund. Mol. Mech. Mutagenesis.

[46] Shen L, Cui J, Liang S, Pang Y, Liu P (2013). Update of research on the role of EZH2 in cancer progression. Onco Targets Ther..

[47] Suvà M-L (2009). EZH2 is essential for glioblastoma cancer stem cell maintenance. Cancer Res..

[48] Zhang X, Ma X, Wang Q, Kong Z (2022). EZH2 targeting to improve the sensitivity of acquired radio-resistance bladder cancer cells. Transl. Oncol..

[49] Gan L (2018). Epigenetic regulation of cancer progression by EZH2: from biological insights to therapeutic potential. Biomark. Res..

[50] Liao Y (2022). Inhibition of EZH2 transactivation function sensitizes solid tumors to genotoxic stress. Proc. Nat. Acad. Sci..

[51] Stricher F, Macri C, Ruff M, Muller S (2013). HSPA8/HSC70 chaperone protein. Autophagy.

[52] Mayer MP, Bukau B (2005). Hsp70 chaperones: Cellular functions and molecular mechanism. Cell Mol. Life Sci..

[53] Jose LHS (2020). Modest declines in proteome quality impair hematopoietic stem cell self-renewal. Cell Rep..

[54] Radman M (2016). Protein damage, radiation sensitivity and aging. DNA Repair.

[55] Kawamura T (2022). VGLL3 increases the dependency of cancer cells on de novo nucleotide synthesis through GART expression. J. Cell Biochem..

[56] Zhou W (2020). Purine metabolism regulates DNA repair and therapy resistance in glioblastoma. Nat. Commun..

[57] Lindell Jonsson E (2019). Exploring radiation response in two head and neck squamous carcinoma cell lines through metabolic profiling. Front. Oncol..

[58] Karigane D (2016). p38α activates purine metabolism to initiate hematopoietic stem/progenitor cell cycling in response to stress. Cell Stem Cell.

[59] Miller KM (2010). Human HDAC1 and HDAC2 function in the DNA-damage response to promote DNA nonhomologous end-joining. Nat. Struct. Mol. Biol..

[60] Groselj B, Sharma NL, Hamdy FC, Kerr M, Kiltie AE (2013). Histone deacetylase inhibitors as radiosensitisers: Effects on DNA damage signalling and repair. Br. J. Cancer.

[61] Wanior M, Krämer A, Knapp S, Joerger AC (2021). Exploiting vulnerabilities of SWI/SNF chromatin remodelling complexes for cancer therapy. Oncogene.

[62] Yamashita M, Suda T (2021). Low-dose ionizing radiations leave scars on human hematopoietic stem and progenitor cells: The role of reactive oxygen species. Haematologica.

[63] Anderson GA, Rodriguez M, Kathrein KL (2020). Regulation of stress induced hematopoiesis. Curr. Opin. Hematol..

[64] Yamaguchi M, Kashiwakura I (2013). Role of reactive oxygen species in the radiation response of human hematopoietic stem/progenitor cells. PLoS ONE.

