# Supplementary material for: Prediction of hub genes and key pathways associated with the radiation response of human hematopoietic stem/progenitor cells using integrated bioinformatics methods
Source: Sci Rep. 2023 Jul 4;13:10762. doi: 10.1038/s41598-023-37981-6 (PMC10319889; doi:10.1038/s41598-023-37981-6)
Supplement: Supplementary file 1 — Supplementary Figures. [file 41598_2023_37981_MOESM1_ESM.pdf]

Prediction of hub genes and key pathways associated with the radiation response of human hematopoietic stem/progenitor cells using integrated bioinformatics methods

Yoshiaki Sato[1], Hironori Yoshino[1], Junya Ishikawa[2], Satoru Monzen[1], Masaru Yamaguchi[1] and Ikuo Kashiwakura[1*]

[1] Department of Radiation Science, Hirosaki University Graduate School of Health Sciences, Hirosaki, Aomori 036-8564, Japan

[2] Department of Medical Radiologic Technology, Faculty of Health Sciences, Kyorin University, Mitaka, Tokyo 181-8612, Japan

*** Correspondence:**
Ikuo Kashiwakura
ikashi@hirosaki-u.ac.jp

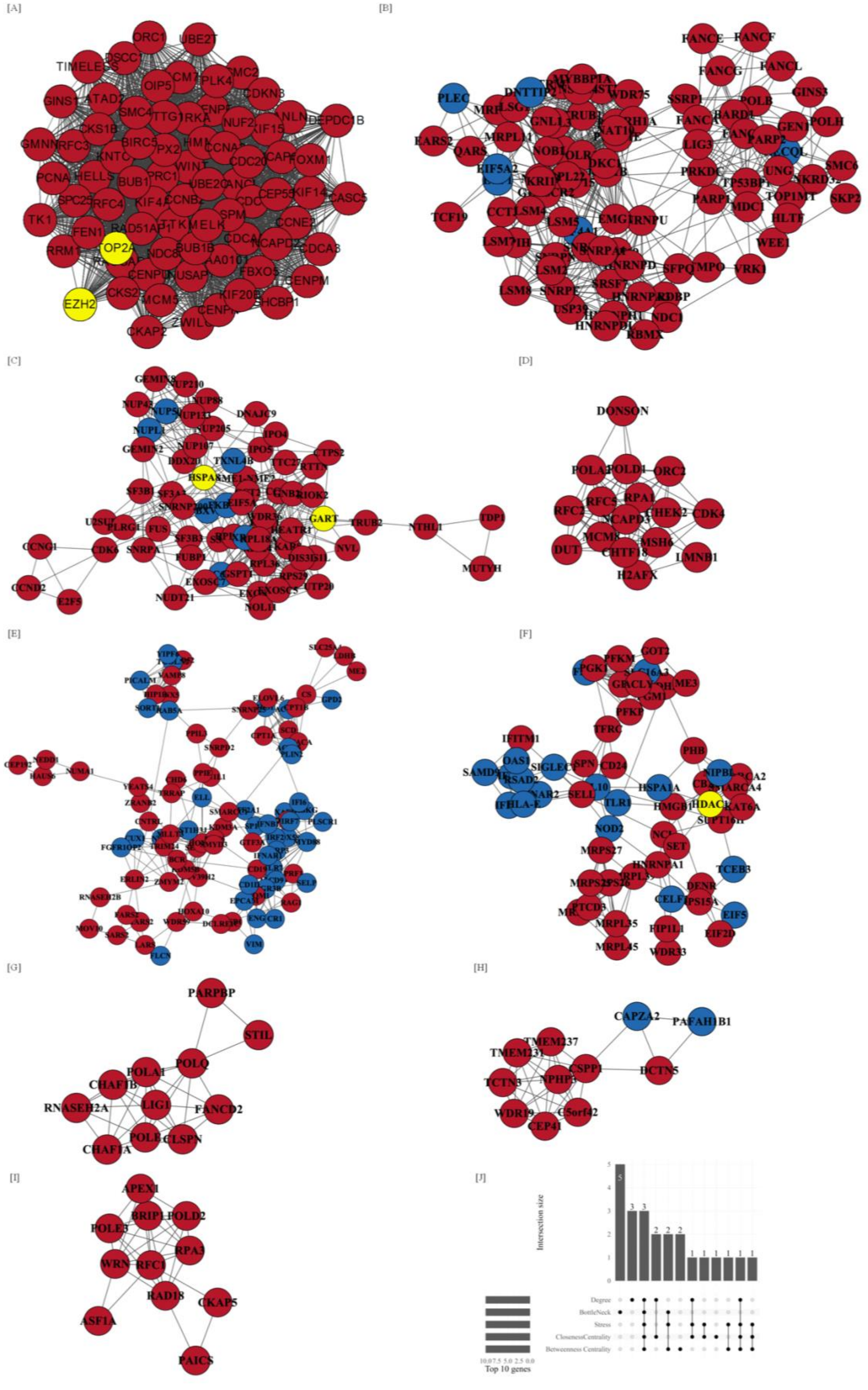
[A]
[B]
[C]
[D]
[E]
[F]
[G]
[H]
[I]
[J]
EZH2
DONSON
POLA1
POLD1
ORC2
RFC2
RFC5
RPA1
CHEK2
CDK4
NCAPD3
MCM8
DUT
CHTF18
MSH6
LMNB1
H2AFX
PARPBP
STIL
POLQ
CHAF1B
LIG1
RNASEH2A
FANCD2
POLE
CLSPN
CHAF1A
CAPZA2
PAFAH1B1
TMEM237
TMEM231
CSPP1
DCTN5
TCTN3
NPHP3
WDR19
C5orf42
CEP41
APEX1
BRIP1
POLD2
POLE3
RPA3
WRN
RFC1
RAD18
CKAP5
ASF1A
PAICS
Intersection size
5
3
3
2
2
2
1
1
1
1
1
1
Degree
BottleNeck
Stress
ClosenessCentrality
Betweenness Centrality
10.0 7.5 5.0 2.5 0.0
Top 10 genes

Supplementary Figure 1. Clusters of cytokine-regulated DEGs and the intersection of topological analysis. (**A-I**) PPIs with cytokine-regulated radiation-induced DEGs were analyzed using the STRING database. All clusters were organized by the Prefuse Force Directed Layout in Cytoscape. Up-regulated DEGs are represented by red color nodes and down-regulated DEGs are represented by blue color nodes. (**J**) The intersection size of the top 10 scored genes between each topological analysis is shown as an upset diagram.

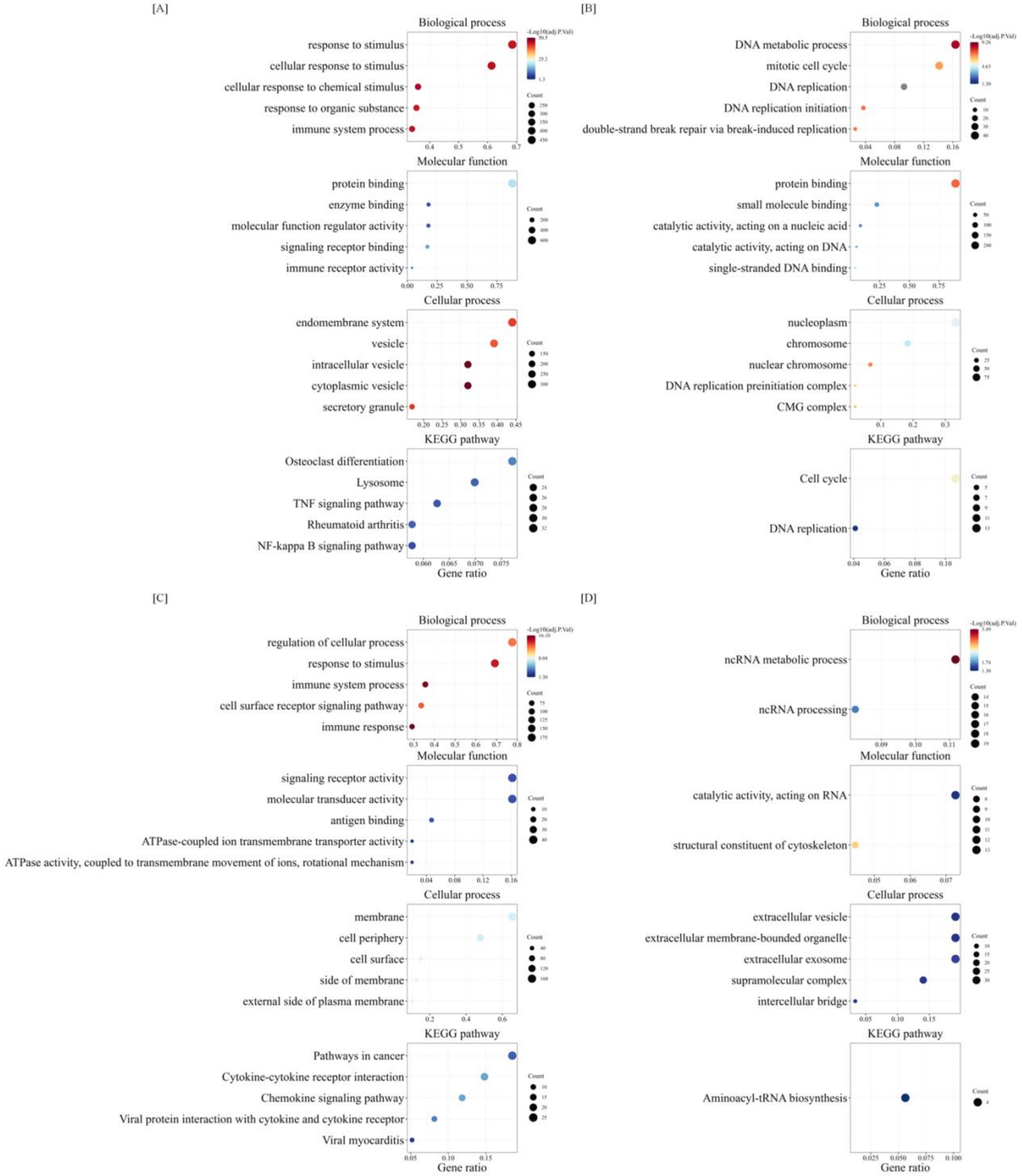

Supplementary Figure 2. GO and KEGG functional enrichment analysis with (**A**) commonly down-, (**B**) commonly up-regulated, (**C**) cytokine-nonresponsive down-, or (**D**) cytokine-nonresponsive up-regulated DEGs. Bubble plots of the top 5 terms and pathways in biological process, cellular component, molecular function, and KEGG pathway analysis based on adjusted p-value are shown. Gene ratio is defined as the ratio between intersection size and query size. The number refers to interaction size, i.e., the number of genes corresponding to an ontology term.

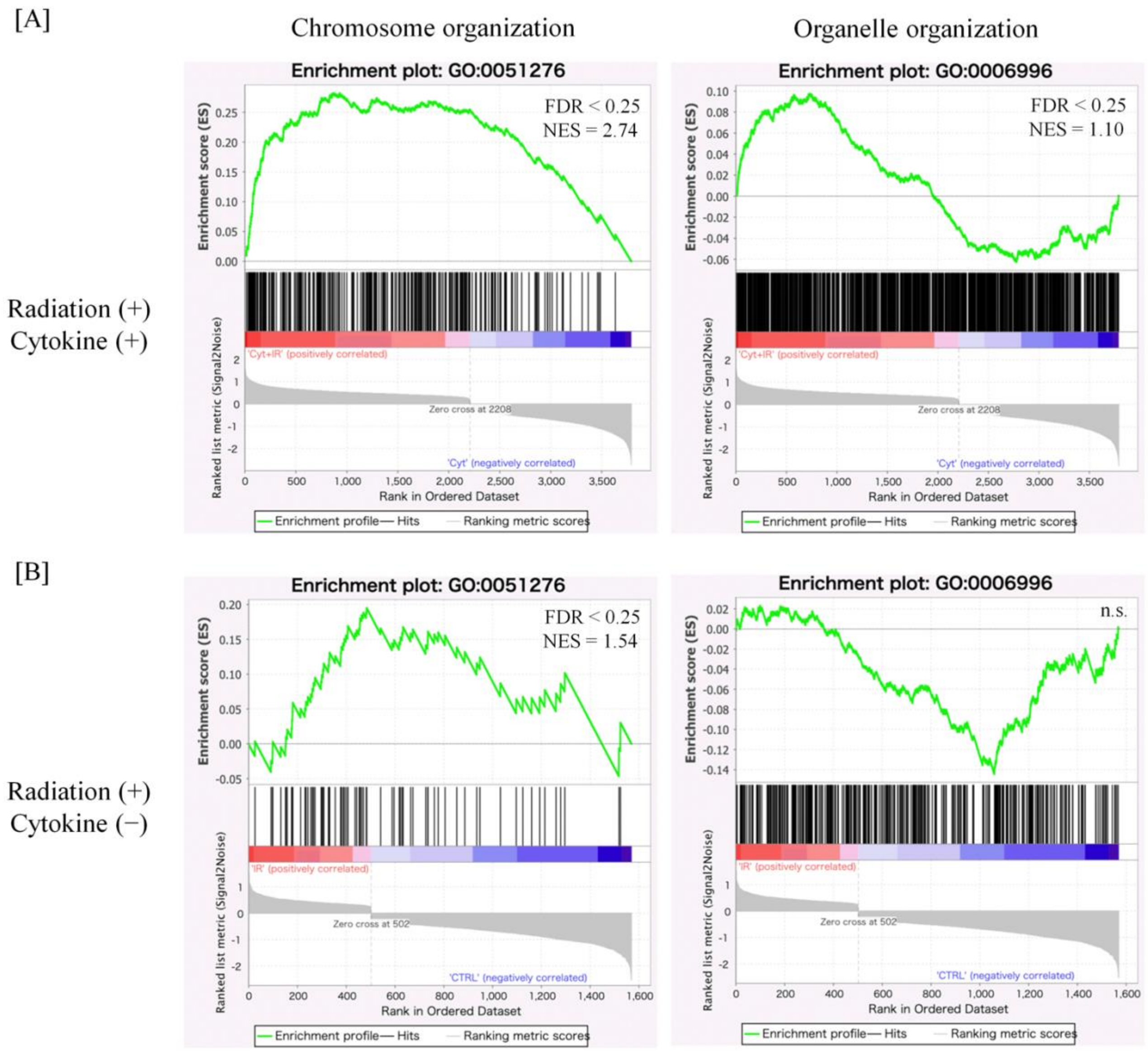

Supplementary Figure 3. Enrichment of genes related to chromosome and organelle organization in human HSPCs co-treated by cytokine and radiation. (**A-D**) The y-axis represents enrichment score (ES) and on the x-axis are genes (vertical black lines) represented in gene sets. The green line connects points of ES and genes. ES is the maximum deviation from zero as calculated for each gene going down the ranked list, and represents the degree of over-representation of a gene set at the top or the bottom of the ranked gene list. The colored band at the bottom represents the degree of correlation of genes with the irradiated phenotype (red for positive and blue for negative correlation). Gene sets were significant only in with or without cytokine treatment. n.s., not significant. NES, normalized ES.
